# Autism spectrum disorder: A mitochondrial disorder

**DOI:** 10.22037/ijcn.v16i2.33066

**Published:** 2021

**Authors:** Josef FINSTERER

**Affiliations:** 1 Messerli Institute, Klinik Landstrasse, Vienna, Austria

**Keywords:** MTDNA, Mitochondrial, Respiratory Chain, Autism, Abnormal behaviour

**Letter to the Editor**

With interest, we read the review article by Ahmadabadi et al. on autism spectrum disorders (ASDs) in inborn errors of metabolism (IEM) (1). They reviewed 37 studies, and found that IEMs underlie autistic features in <5% of the patients, and that there is growing evidence on the association between ASDs and mitochondrial disorders (MIDs), including mitochondrial encephalopathy, lactic acidosis, stroke-like episodes (MELAS) syndrome, and respiratory chain complex III/IV deficiency (1). The authors concluded that the syndromic autism, a strong family history, or consanguinity suggest IEM (1). However, we have raised the following comments and concerns:

We disagree with the idea that mitochondrial disorders (MIDs) have only two points of onset as they can occur at any age, and their broad variability, even in a family, results from the peculiarities of mitochondrial genetics. Due to mutations in mtDNA-located genes, MIDs are maternally transmitted, and the underlying mtDNA mutations may not occur in each mtDNA copy (heteroplasmy). Moreover, mtDNA copy number may considerably vary from one mitochondrion to another mitochondrion, particularly if the mutation is located in a nuclearly-encoded gene. MIDs frequently manifest in the central nervous system (CNS), and the CNS manifestations may include psychiatric or neurological diseases or both. The psychiatric diseases range from mild cognitive impairment and personality change to delirium and psychosis. Autism has been frequently reported in MIDs (2) and may or may not be associated with cerebral morphological alterations. 

We also disagree that ASD occurs only in MELAS and complex-III/IV deficiency (1). ASD has also been reported in a patient carrying the mtDNA variant m.8363G>A, whose sister was carrying the same variant and presented with Leigh syndrome (3), as well as in two Korean siblings carrying the variant c.790C>T in *TFB2M* (4). In a study on 60 ASD patients, single mtDNA deletions were detected in 16.6% of the patients (5). In the same study, the ten patients with mtDNA deletions also carried single nucleotide variants (SNVs) in ASD-associated genes (5). In a study on 95 ASD patients, the mtDNA content decreased in the ASD patients, and 49 putative pathogenic mtDNA variants were detected (6). In a study on 10 families with ASD, whole-exome sequencing revealed the variants of interest (VOIs) in the *ND5* gene in one family and VOIs in *ATP6* and *NDUFS4* in another family (7). In a study on 24 Iranian ASD patients, mtDNA mutations 16126T>C, m.14569G>A, and m.,1811A>G, all of which were located in non-coding regions, showed a significant relationship with ASD (8).

In general, there is ample evidence indicating that the IEM, which is most frequently associated with ASD, is MID. According to the literature, the mtDNA variants but not the nDNA variants have been more frequently associated with ASD. Patients with an ASD should be first examined in terms of the presence or absence of MID.
